# Profiling of exosomal microRNAs expression in umbilical cord blood from normal and preeclampsia patients

**DOI:** 10.1186/s12884-022-04449-w

**Published:** 2022-02-14

**Authors:** Hai-Tao Pan, Xiao-Liang Shi, Min Fang, Xiang-Mei Sun, Pan-Pan Chen, Jin-Long Ding, Gui-Yu Xia, Bin Yu, Tao Zhang, Hong-Dan Zhu

**Affiliations:** 1 Shaoxing Maternity and Child Health Care Hospital, Shaoxing, 312000 China; 2 Obstetrics and Gynecology Hospital of Shaoxing University, Shaoxing, China; 3 The International Peace Maternity and Child Health Hospital, School of Medicine, Shanghai Jiao Tong University, Shanghai, 200030 China

**Keywords:** Preeclampsia, Umbilical serum, Exosomal miRNAs, Offspring, Microvascular dysfunction, Gamete and embryo-fetal origins of adult diseases

## Abstract

**Background:**

Epidemiological and experimental studies suggest that preeclampsia has a negative impact on maternity and offspring health. Previous studies report that dysregulation in utero-environment increases risk for elderly disease such as cardiovascular disease. However, the underlying mechanisms remain elusive. Specific microRNAs (miRNAs) are packaged in exosomes may regulate microvascular dysfunction in offspring of mothers with preeclampsia. The present study aimed to identify the differential expression profiles of microRNAs in the serum exosomes between patients with preeclampsia and normal pregnancies.

**Methods:**

A comprehensive miRNA sequence-based approach was performed to compare exosomes carry miRNAs (Exo-miRNAs) expression levels in umbilical serum between normal and preeclampsia patients. Exosomes were isolated using the ExoQuick precipitation kit. Serum exosomes were then viewed under electron microscopy, and their characteristics determined by western blotting and nanoparticle-tracking analysis. Illumina platform was used to perform sequencing. Bioinformatics analysis was used to explore differentially expressed Exo-miRNAs in umbilical serum.

**Results:**

Based on sequence similarity, 1733 known miRNAs were retrieved. Furthermore, 157 mature miRNAs in serum exosomes were significantly differential expressed between PE and those control groups (*P*<0.05, log_2_|FC| > 1). Out, of the 157 miRNAs, 96 were upregulated miRNAs whereas 61 miRNAs were downregulated. The 157 differentially expressed miRNAs targeted 51,424 differentially expressed genes. Functional analysis through KEGG pathway and Gene Ontology results uncovered that target genes of miRNAs with differential expression were significantly linked to several pathways and biological processes.

**Conclusion:**

The findings of this study showed differential expression of umbilical serum Exo-miRNAs in normal compared with PE patients, implying that these Exo-miRNAs may associate with microvascular dysfunction in offspring of mothers with preeclampsia.

**Supplementary Information:**

The online version contains supplementary material available at 10.1186/s12884-022-04449-w.

## Background

Preeclampsia (PE) is a complication of pregnancy. PE is associated with initiation of hypertension after 20 weeks of gestation accompanied by proteinuria, and affects about 5% of pregnant mothers [1]. Previous studies report that women with PE are likely to develop cardiovascular diseases [2]. In addition, offspring of mothers with preeclampsia have higher risk of cardiovascular disease [3].

Exosomes are membrane-bound vesicles released from all cell types into body fluids including plasma, urine, saliva and malignant effusions [4]. The size of exosomes ranges between 30 and 150 nm in diameter. Studies on exosomes show that they are implicated in modulating intercellular communication [5]. Exosomes secrete substances such as proteins, lncRNA, mRNA and microRNAs that are involved in regulation cell function [6]. Epigenetic modifications including alterations in microRNA levels are implicated in development of cardiovascular disease [7]. MicroRNAs (miRNAs) are involved in the modulation of key cellular activities, e.g., proliferation, metabolism, immune activities, apoptosis, neurodevelopment and epigenetics [8]. Impaired maternal cardiovascular function (cerebrovascular and hidden cardiovascular diseases) and dysfunctional placenta may cause epigenetic changes due to pregnancy-related complications in umbilical cord blood. These changes may increase risk of cerebrovascular and cardiovascular diseases in the newborn [9]. The ‘Gamete and Embryo-fetal Origins of Adult Diseases’ hypothesis states that patterns of fetal growth and development are implicated in risks to chronic non-communicable diseases in adult life [10]. However, development of altered vascular function and endothelial dysfunction in offspring has not been fully explored.

Previous studies report that PE increases risk of cardiovascular disease in pregnant mothers [11].In addition, PE causes persistent vascular dysfunction in pulmonary and systemic circulation of the offspring [12]. However, studies have not carried out high throughput sequencing of microRNAs in umbilical cord blood exosomes in women with or without preeclampsia have not been attempted. The current study sought to generate an exosomal microRNAs map of umbilical cord blood in preeclampsia.

## Materials & methods

### Study participants

Umbilical serum from normal and mild preeclampsia patients were collected after cesarean deliveries at the Shaoxing Maternity and Child Health Care Hospital. The Ethics Committee of Shaoxing Maternity and Child Health Care Hospital approved the study (Ethical approval no. 2018022). All subjects enrolled in this study provided a written informed consent. Umbilical sera were collected from healthy individuals (*n* = 5) and PE patients (*n* = 5). Inclusion criteria for the study were: singleton pregnancy, full-term delivery, maternal age between 25 and 35 years, diastolic blood pressure ≥ 90 mmHg and/or systolic blood pressure ≥ 140 mmHg, normal birth weight ranging between 2500 and 4000 g, no known birth or other complications. Normal control subjects were matched with PE women for maternal age, gestational weeks, gravidity and parity. Detailed patient characteristics are further outlined in Table 1.

**Table 1:** The clinical data of the patient and control groups

	Sample name	Maternal age (y)	Gestational age (wk)	Birth weight (g)	Gender	Maternal systolic/diastolic blood pressure (mm Hg)	Proteinuria	Maternal fasting blood glucose (mmol/L)	Diagnosis
Control	C1	27	38	3600	M	119/73	–	4.88	Normal
	C2	30	37	3500	M	110/68	–	4.93	Normal
	C3	29	39	3400	M	123/75	–	4.91	Normal
	C4	28	37	3650	F	125/76	–	5.03	Normal
	C5	28	40	3550	F	112/73	–	4.86	Normal
Preeclampsia	T1	28	38	3450	M	152/86	++	5.08	Preeclampsia
	T2	30	37	3300	M	149/78	++	5.03	Preeclampsia
	T3	31	37	3250	M	150/75	++	4.96	Preeclampsia
	T4	27	37	3400	F	156/94	++	5.01	Preeclampsia
	T5	29	38	3200	F	149/88	++	4.91	Preeclampsia

### Exosomes purification and characterization

Samples were sieved using 0.22-μm-pore-size filters following a method describedpreviously [13]. After precipitation of exosomes with the ExoQuick Exosome Precipitation Solution (System Biosciences-SBI), they were analyzed using an electron microscope (FEI Quanta 250, Thermo Fisher Scientific, Eindhoven, the Netherlands), the Nano particle tracking analysis (NTA) (ZetaView PMX 110, Particle Metrix, Meerbusch, Germany), and Western blot analysis (for CD63) were used to characterize isolated exosomes.

### miRNA sequencing of exosomes and sequence analysis

Human umbilical serum samples were obtained from 10 female volunteers (5 mild preeclampsia patients and 5 healthy volunteers). Illumina TruSeq RNA Sample Preparation Kit (catalog # RS-122-2001) was used for library preparation. Illumina HiSeq X Ten platform was use to sequence libraries to obtain 150 bp paired-end data (minimal depth of 30× base coverage). CASAVA tool (v1.8 Illumina, San Diego, CA, USA) was used to obtain “Fastq.File”. Raw reads counts were normalized as Reads Per Kilobase of transcript per Million mapped reads (RPKM).

miRNA sequences were analyzed using miRWalk (http://mirwalk.uni-hd.de) and miRBase 22.0 (http://www.mirbase.org) webservers FastQC tool was used to perform quality control of sequence reads (http://www.bioinformatics.babraham.ac.uk/projects/fastqc/). pheatmap package in R was used to generate heatmaps. RNAhybrid (energy <− 25) and miRanda (energy <− 20, score ≥ 150) tools were used predict potential miRNA targets. Significant protein expression, gene ontology (GO) and associated pathway were explored through bioinformatics analyses. Kyoto Encyclopedia of Genes and Genomes (KEGG) pathway and GO analyses were carried out using DAVID 6.8 tool (https://david.ncifcrf.gov) [14–17].

### Western blotting analysis

Western blotting was carried out to quantify protein levels as previously reported [18]. Protein samples were subjected to 10% SDS-PAGE gel electrophoresis. After transfer to membranes, the proteins were probed with the following primary antibodies (CD63 (SBI EXOAB-CD63A-1, Mountain View, CA, USA, 1:1000), GAPDH (Beyotime AG019–1, Shanghai, China, 1:1000) and secondary antibody (1: 1000). The proteins were visualized using the Minichemi Chemiluminescence Imaging Instrument (Sage, Beijing, China). This assay was carried out in triplicates.

### Data analysis

The results are presented as means and standard deviations (SD) obtained from three experimental repeats, unless otherwise stated. Group comparisons were done using Student’s t-test. *P* < 0.05 indicated significant differences.

## Results

### Extraction and analysis of serum-derived EXOs

**Fig. 1:**
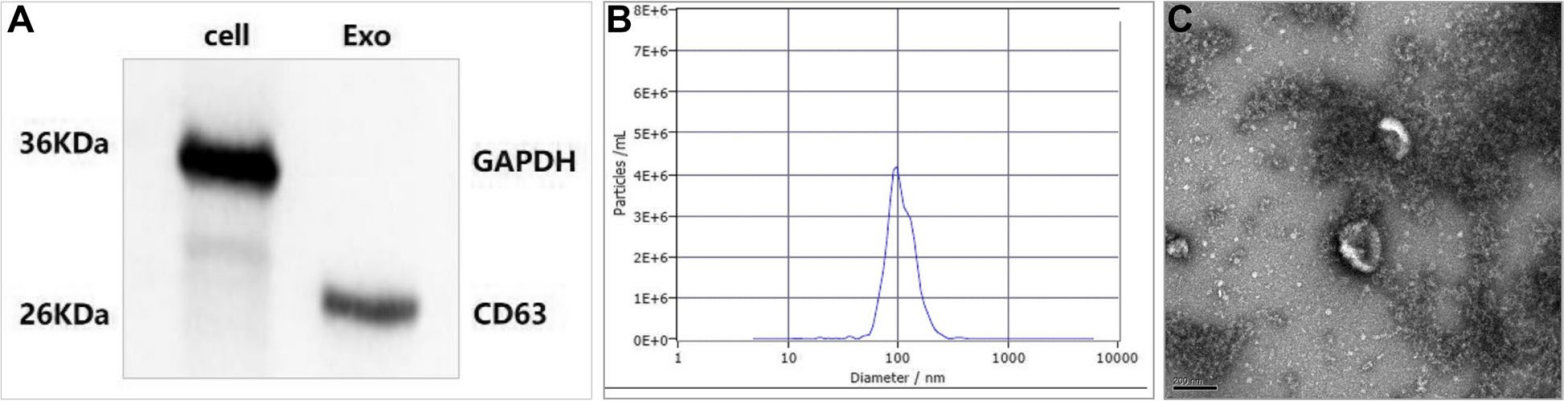
Characteristics of human serum exosomes. **A** Western blot analysis of exosomes surface markers (CD63). **B** Representative nanoparticle tracking analysis (NTA) profile human serum exosomes **C** Serum-derived exosomes as observed under electron microscope (scale bar, 200 nm)

First, exosomes were isolated and their baseline characteristics were evaluated (Fig. 1). Presence of tetraspanin CD63 (Fig. 1A, left panel) implies that isolated vesicles were mainly exosomes. Characterization of these purified exosomes by NTA showed that their size ranged between ~ 100.5 ± 4.0 (Fig. 1B). This finding matched with typical sizes of exosomes. Additionally, the morphology and size of the exosomes were also examined by TEM (scale bar, 200 nm) (Fig. 1C).

### Analysis of differential expression of serum miRNAs in serum EXOs

**Fig. 2:**
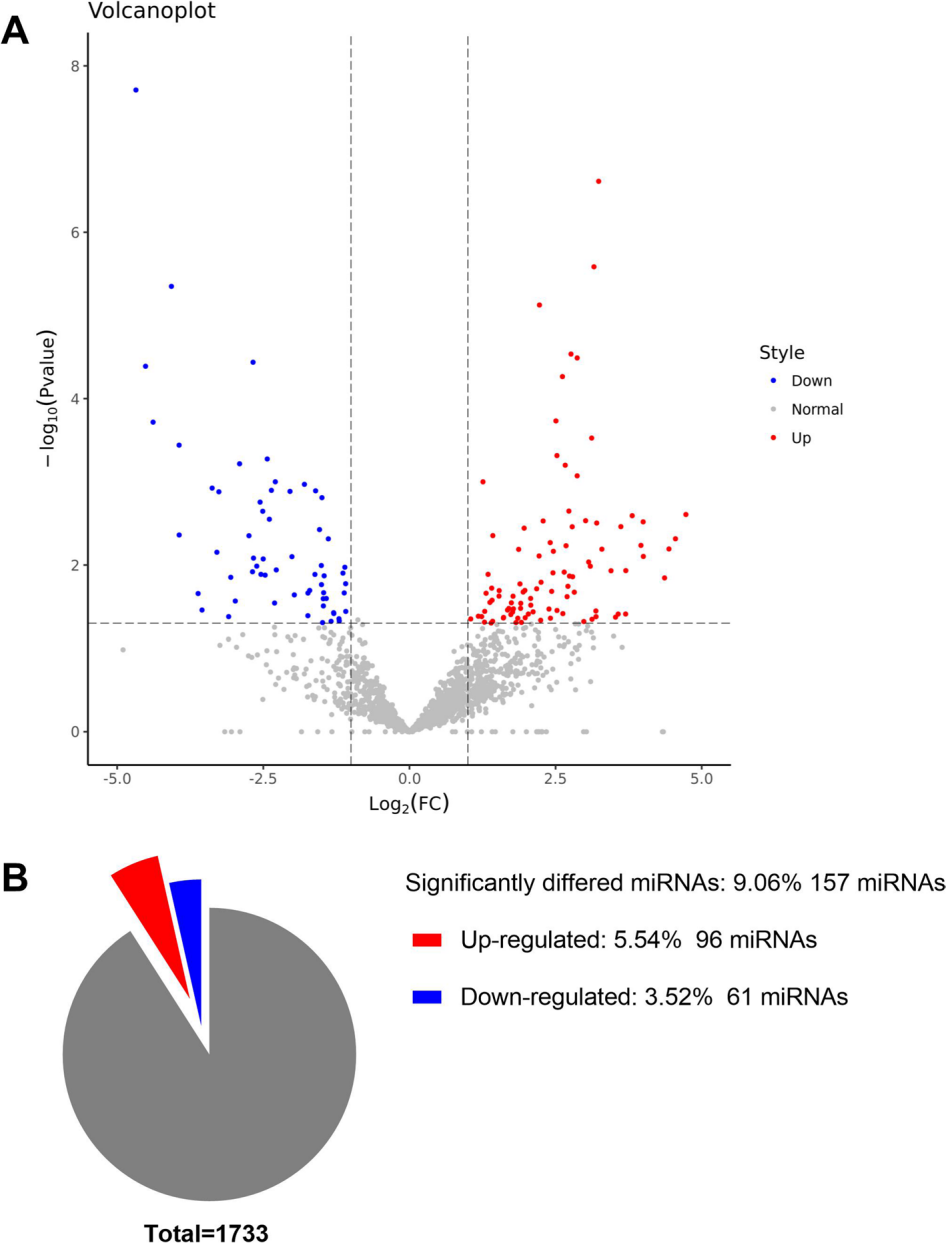
Cluster analysis of differentially expressed exosomal miRNAs isolated from serum of PE patients and normal controls. **A** Heat map showing hierarchical clustering (HCL) of normalized levels of the 157 differentially expressed microRNAs from PE (*n* = 5) and normal control (*n* = 5). **B** Volcano map showing distribution of differentially expressed microRNAs based on the *p* values and fold-changes. *p* < 0.05 and |log 2(fold-change) | ≥1 were used to show differential expression

A total of 1733 known miRNAs were identified based on sequence similarity (Supplemental Table S1). The original sequencing data can be accessed on the NCBI SRA database: PRJNA706741. There were 157 mature miRNAs that were differentially expressed between samples from PE and control groups (log_2_|FC| > 1, *P*<0.05; Supplementary Table S2). Out of the 157 differentially expressed miRNAs, 96 were upregulated whereas 61 were downregulated and were presented in a volcano plot (Fig. 2).

### GO and pathway analyses for miRNA target genes

**Fig. 3:**
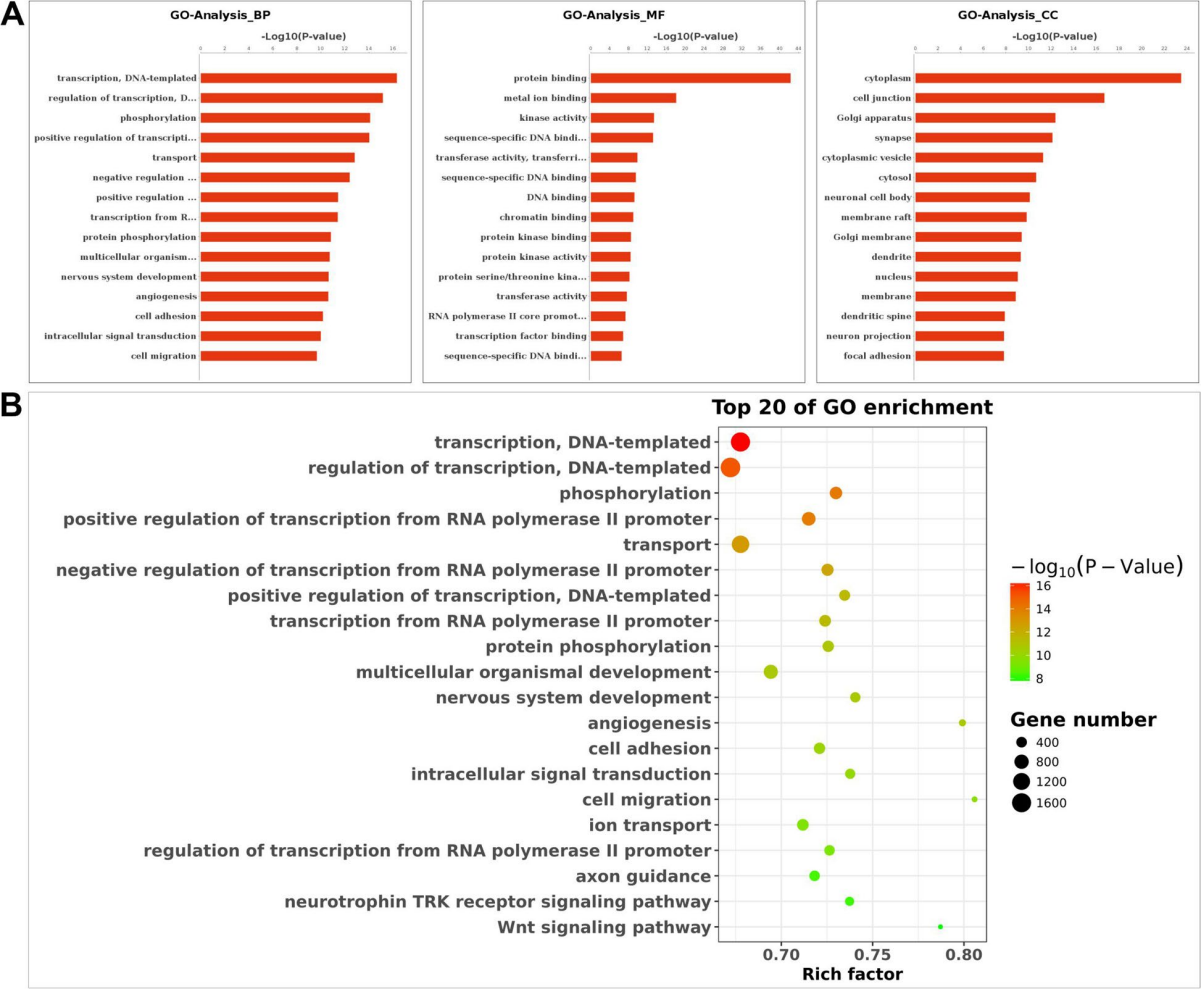
Gene ontology (GO) enrichment analysis of predicted target genes. **A** GO analysis of differentially expressed genes for Biological Process (BP), Molecular Function (MF), and Cellular Component (CC) terms

**Fig. 4:**
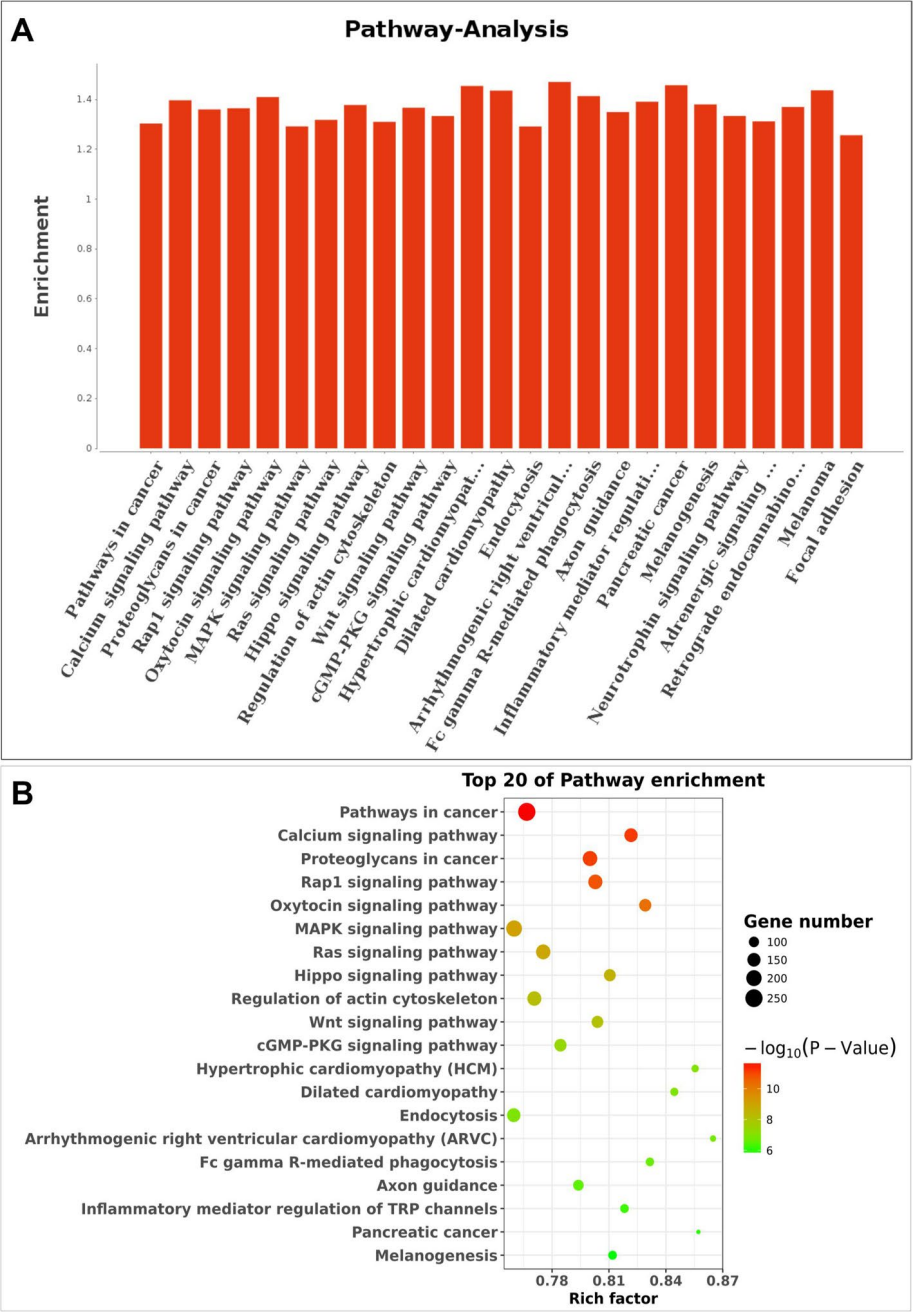
KEGG pathway analysis for predicted target genes of differentially expressed miRNAs. **A** Coordinate axis X: -log_10_ (*P*-value); coordinate axis Y: Pathway-Term entry name. **B** Coordinate axis X: -log_10_ (*P*-Value); coordinate axis Y: Pathway-Term entry name

Analysis of the 157 differentially expressed miRNAs showed that they can target 51,424 differentially expressed genes (Fig. S1, Supplemental Table S3). In addition, GO (Fig. 3) and KEGG pathway analyses (Fig. 4) were conducted to explore the roles of miRNA target genes. Results showed that the genes were linked to transcription, phosphorylation, development and angiogenesis. KEGG results revealed that the genes were linked to rap1 pathways, oxytocin pathways, MAPK and calcium signaling pathways.

## Discussion

Preeclampsia is a complex pregnancy-related syndrome involving several factors and is linked to high risk of cardiovascular disease in pregnant mothers and their newborns [23]. In a previous study on preeclampsia patients, we carried out iTRAQ analysis on umbilical artery samples. The findings showed 53 differentially expressed proteins in which participate in developmental processes of the cardiovascular system. Overall, 1733 known miRNAs were found in this study. Analysis of exosomal microRNAs sequence data showed 157 differentially expressed mature miRNAs in PE-derived serum exosomes compared with control group. Out of the 157 differentially expressed miRNAs, 96 were upregulated whereas 61 miRNAs were downregulated. Further analysis showed that the 157 differentially expressed miRNAs targeted 51,424 DEGs. Gene Ontology and KEGG pathway analyses showed significant enrichment of the target DEGs in several biological processes and pathways.

Umbilical cord blood is characterized by specific features compared with adult blood, therefore, studies explore it to understand the fetal state. In the present study, several significantly differentially expressed miRNAs related with cardiovascular function. miR-483-5p associates with obesity and cardiovascular disease [19], also ameliorates hypercholesterolemia by inhibiting PCSK9 production [20]. The miRNA miR-483-3p is an important modulator of endothelial integrity in type 2 diabetes (T2D) patients and is a potential therapeutic target for alleviating endothelial regeneration after injury T2Dpatients [21]. hsa-miR-1237-3p expression is correlated with certain fatty acids levels [22]. hsa-miR-365b-5p and hsa-miR-155-5p affectively modulates vascular functions and proliferation of HUVEC [19]. Moreover, hsa-miR-155-5p is a potential biomarker which for differentiating Hypertrophic cardiomyopathy (HCM) patients from healthy controls [23]. Changes in expression levels of miR-200b in acute hypoxia may exert a proangiogenic effect by downregulating Klf2 thus stabilizing HIF-1 signaling [24]. hsa-miR-200b-3p can be used as treatment targets for hypoxic-related and cardiovascular diseases including different cancer types [25]. hsa-miR-342-3p is a potential biomarker in children with endothelial dysfunction [26]. Notably, hsa-miR-140-3p expression is downregulated in CAD (coronary artery disease) patients [27]. hsa-miR-3909 is correlated with rheumatic valvular heart disease [28]. However, the roles of different miRNA expressions in offspring microvascular dysfunction of preeclampsia remains have not been explored.

Several DEGs were identified that are targeted by differentially expressed miRNAs. GO and KEGG pathway analyses were used to explore potential biological functions and potential mechanisms of miRNA found in offspring microvascular dysfunction of preeclampsia. GO term analysis of these target genes showed a number of remarkably linked to GO terms associated with transcription, phosphorylation, development and angiogenesis. Multiple genetic disorders, mainly metabolic and cardiovascular diseases are associated with transcription of metabolism-associated genes [29]. Protein phosphorylation is a key signaling mechanism for regulating cellular pathways implicated in several physiological processes associated with cardiovascular and metabolic systems in the body [30]. Recently, epigenetics especially microRNAs have attracted growing attention in regulating embryonic vascular development [31]. Exosomal microRNAs are implicated in modulating endothelial cell function and angiogenesis [32].

Findings from KEGG pathway analysis uncovered that the target genes correlated with calcium rap1, oxytocin and MAPK signaling pathways. Calcium signaling plays a key role in cardiovascular excitability and function [33]. High expression levels of endogenous TRPM8 inhibits the function of Rap1 GTPase and plays an important role in modulation of activities of vascular endothelial cells by inhibiting migration [34]. Oxytocin also exerts cardiovascular and antiobesity effects through central and peripheral oxytocin receptor [35]. Crosstalk between p38 MAPK signaling and extracellular signal-related kinase (ERK) ½ modulates various physiological functions, such as cardiac fibrosis and vascular smooth muscle cell migration [36].

## Conclusions

Our findings, indicating the differential expression of umbilical serum Exo-miRNAs between normal and PE patients, reveal that these Exo-miRNAs may associate with microvascular dysfunction in offspring of mothers with preeclampsia.

## Supplementary Information

**Additional file 1: Figure S1.** Venn diagram of target genes of differentially expressed miRNAs. RNAhybrid: Energy <− 25. Miranda: score > 150, Energy <− 20.

**Additional file 2: Figure S2.** Original western blot gels for Fig. 1A.

**Additional file 3: Table S1.** A total of 1733 miRNAs were identified in serum exosomes from patients with PE and control groups.

**Additional file 4: Table S2.** 157 differentially expressed miRNAs in serum exosomes between the PE group and the control group.

**Additional file 5: Table S3.** Target genes of differentially expressed miRNAs.

## Data Availability

The raw sequence reads are available under the SRA accession numbers PRJNA706741 [https://www.ncbi.nlm.nih.gov/sra/PRJNA706741].

## Abbreviations

PE: Preeclampsia
miRNAs: microRNAs
GO: Gene ontology
KEGG: Kyoto Encyclopedia of Genes and Genomes
