# Supplementary figures and images for: Profiling of exosomal microRNAs expression in umbilical cord blood from normal and preeclampsia patients
Source: BMC Pregnancy Childbirth. 2022 Feb 14;22:124. doi: 10.1186/s12884-022-04449-w (PMC8842963; doi:10.1186/s12884-022-04449-w)

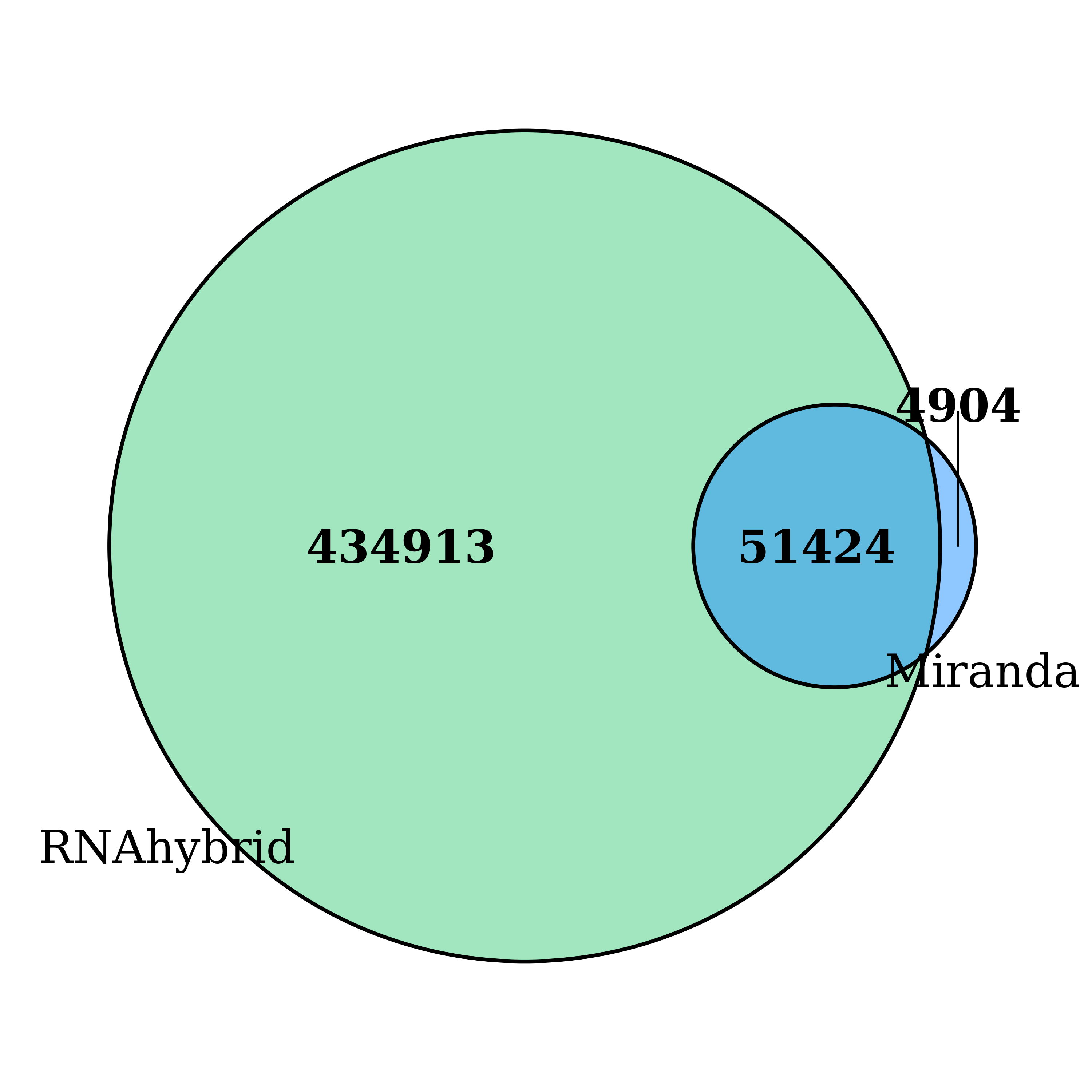

Supplement: Supplementary file 1 — Additional file 1: Figure S1. Venn diagram of target genes of differentially expressed miRNAs. RNAhybrid: Energy <− 25. Miranda: score > 150, Energy <− 20. [file 12884_2022_4449_MOESM1_ESM.png]

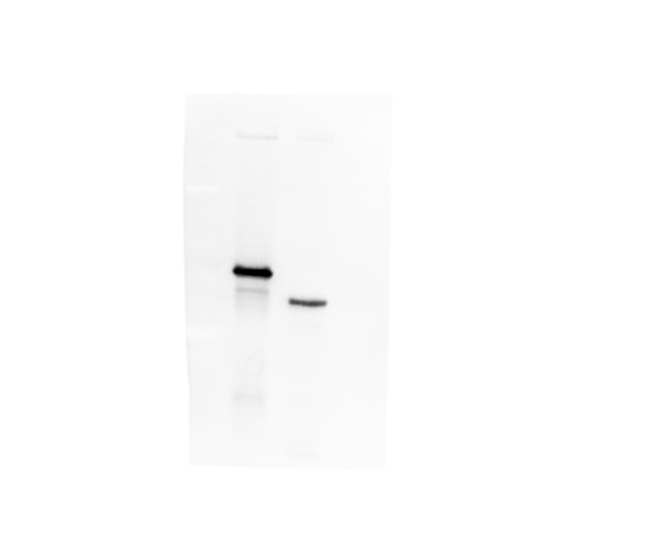

Supplement: Supplementary file 2 — Additional file 2: Figure S2. Original western blot gels for Fig. 1A. [file 12884_2022_4449_MOESM2_ESM.tif]
